# Transcallosal approach for a giant third ventricle craniopharyngioma: illustrative case

**DOI:** 10.3171/2026.7.FOCVID2686

**Published:** 2026-10-01

**Authors:** Kyohei Yamashiro, Kentaro Watanabe, Nobuyuki Watanabe, Ryo Ishikawa, Yumi Matsuzawa, Kyoichi Tomoto, Kohei Ishikawa, Michihiro Tanaka, Yuichi Murayama

**Affiliations:** 1 Department of Neurosurgery, The Jikei University School of Medicine, Minato-ku, Tokyo; and; 2 Department of Neurosurgery, Kameda Medical Center, Kamogawa, Chiba, Japan

**Keywords:** craniopharyngioma, third ventricle, transcallosal approach, 3D surgical simulation

## Abstract

Giant third ventricle craniopharyngiomas pose surgical challenges due to their deep location and critical neurovascular relationships. A 22-year-old woman presented with headache, bitemporal hemianopia, and memory impairment from a giant multicystic craniopharyngioma extending from the third ventricle into the left lateral ventricle with obstructive hydrocephalus. Using GRID 3D simulation, the authors compared four corridors; a transcallosal transforaminal approach was selected based on the enlarged foramen of Monro and medially displaced fornix. Gross-total resection was achieved, with marked visual and memory recovery and return to work. Patient-specific 3D simulation enables objective approach selection. The transcallosal route is effective for retrochiasmatic third ventricle craniopharyngiomas with hydrocephalus.

The video can be found here: https://stream.cadmore.media/r10.3171/2026.7.FOCVID2686

## Transcript

This is a surgical video presentation, the transcallosal approach for a giant third ventricle craniopharyngioma.

### 0:28 Case Presentation.

The patient was a 22-year-old female nurse. She presented with a 1-month history of headache. She gradually developed bitemporal hemianopia, and progressive memory impairment had begun to interfere with her clinical duties.

### 0:47 Preoperative Image.

Gadolinium-enhanced T1-weighted MRI demonstrated a multicystic tumor with calcified components. The optic chiasm was prefixed, with a narrow subchiasmatic corridor. The lesion extended cephalad from the third ventricle into the left lateral ventricle, obstructing the foramen of Monro and causing obstructive hydrocephalus. CT confirmed the calcification: craniopharyngioma. According to the Puget and Sainte-Rose classification, this lesion was categorized as grade 2.

### 1:23 Preoperative Endocrine Profile.

The preoperative endocrine profile showed decreased TSH and LH levels, suggesting partial anterior pituitary dysfunction. Other pituitary hormones were within their normal ranges. Given the compression of the pituitary stalk and the risk of perioperative adrenal insufficiency, hydrocortisone replacement therapy was initiated preoperatively.

### 1:50 Rationale for the Procedure.

Together with the patient’s age, these findings were highly suggestive of an adamantinomatous craniopharyngioma.

Surgical resection was indicated for two reasons: visual field defects from optic chiasm compression and secondary obstructive hydrocephalus.

Our hospital performs preoperative simulations using GRID, a 3D CG simulation software.

### 2:17 Surgical Approach.

Three anatomical factors guided our approach selection: the prefixed chiasm; the tumor size, which made pituitary stalk preservation unlikely; and the already disrupted wall of the third ventricle. Four approaches were considered: transnasal, basal interhemispheric, transcortical, and transcallosal.

### 2:41 Transnasal Transsphenoidal Approach.

The transnasal transsphenoidal route would require pituitary transposition for tumor resection. Dural reconstruction would be challenging. The superior pole of the tumor would be difficult to reach endoscopically.

### 2:59 Basal Interhemispheric Approach.

The basal interhemispheric approach provides a wide field through broad opening of the interhemispheric fissure. However, it is technically demanding and requires an extensive craniotomy. Bilateral olfactory nerves are at risk, frequently resulting in postoperative anosmia, and the genu of the corpus callosum can limit superior tumor exposure. Superior pole of the tumor would also be difficult to reach.

### 3:32 Transcallosal Approach.

The transcallosal approach allows the ventricle to be reached through only a 2-cm callosotomy. On the simulated intraventricular view, the anterior septal vein was clearly visualized, the foramen of Monro appeared enlarged, and the fornix was displaced medially. Based on these findings, the transforaminal approach was selected. The vertically elongated tumor extended through the foramen of Monro, providing a straight trajectory from the callosotomy to the pituitary stalk. The basilar artery and the surrounding peripituitary structures were anticipated to become visible.

### 4:22 Transcortical Approach Versus Transcallosal Approach.

Transcortical resection could similarly achieve gross-total resection. The transcallosal approach was preferred for its reduced damage to the normal brain parenchyma, the more natural surgical trajectory as illustrated, and improved maneuverability within the deep working space. We therefore selected the transcallosal route.

### 4:49 Surgical Video.

The patient was placed in the supine position, and a left-sided bifrontal skin incision was performed. An approximately 5-cm craniotomy was created across the midline. A left interhemispheric approach was used to reach the corpus callosum. Inspection of the left lateral ventricle revealed the anterior septal vein, as predicted by the GRID simulation. A tumor extending from the foramen of Monro into the lateral ventricle was identified. Intraoperative frozen-section diagnosis confirmed an adamantinomatous craniopharyngioma. There was no adhesion between the tumor and the surrounding normal tissue. Tumor debulking was then performed. The posterior portion of the tumor was detached first.

On the medial and anterior aspects, resection was performed more cautiously because the fornix was presumed to be present in this region. Dissection was continued along the tumor capsule.

Additional internal debulking was performed as needed. The lateral portion of the tumor was then detached. The tumor was removed in a piecemeal fashion. At the tumor base, the thalamoperforating artery was identified. The basilar artery apex along with other posterior vascular structures became clearly visible. Gross-total resection was achieved.

The resection cavity was inspected endoscopically. Structures within the lateral ventricle were clearly observed. Through the open third ventricle, the basilar apex and pituitary structures were visualized, matching the GRID simulation.

### 6:41 Postoperative Image.

Postoperative MRI confirmed gross-total resection. Histopathology demonstrated an adamantinomatous craniopharyngioma, positive for p63 and S100, with a Ki-67 labeling index of approximately 5%.

### 6:57 Postoperative Image: Hypothalamic Assessment.

Preoperative and postoperative sagittal and coronal images are compared. According to the Puget and Sainte-Rose classification, which grades hypothalamic involvement by craniopharyngiomas, this case was categorized as grade 2. Fortunately, no hypothalamic symptoms were observed postoperatively

### 7:21 Postoperative Image: Fornix Preservation.

Postoperative heavily T2-weighted MRI confirmed that the fornix was preserved bilaterally, as demonstrated on the axial, coronal, and sagittal images. Although the patient initially experienced short-term memory impairment—likely reflecting transient fornix and hypothalamic manipulation—her cognitive function gradually improved with rehabilitation.

### 7:48 Postoperative Course of Diabetes Insipidus.

Central diabetes insipidus developed in the immediate postoperative period. Oral desmopressin, in the form of Minirin Melt, was administered, and the urine output gradually normalized to physiological levels by the end of the 1st postoperative week.

### 8:10 Postoperative Endocrine Course.

At 6 months postoperatively, panhypopituitarism was confirmed, as expected from the intraoperative sacrifice of the pituitary stalk. All four anterior pituitary axes were suppressed, and prolactin was elevated due to the classic stalk effect. Multihormonal replacement therapy was initiated, including levothyroxine, hydrocortisone, desmopressin, estradiol, dydrogesterone, and somapacitan. Under this regimen, her endocrine status has remained stable.

### 8:48 Postoperative Course.

Overall, the patient recovered well from surgery. Her visual field impairment improved markedly. Short-term memory impairment, which occurred in the early postoperative period, gradually resolved with cognitive rehabilitation. Under adequate hormone replacement, she was able to return to her nursing duties 5 months postoperatively.

### 9:14 Conclusions.

In conclusion, GRID-based preoperative simulation is useful for selecting the optimal surgical approach. Among craniopharyngioma cases, we consider the transcallosal approach to be effective for tumors of the retrochiasmatic type that predominantly extend into the third ventricle and are associated with secondary hydrocephalus. When using a transcallosal approach, the subroute should be carefully selected, with particular attention to the fornix and the distorted ventricular anatomy on preoperative imaging.

### 9:52 References.^1–8^

## Acknowledgments

ElevenLabs version 3 was used to generate the voice-over narration of the video from the author-written English transcript. Genspark Workstation 6.0 was used to assist with translation between Japanese and English for the transcript and abstract text.

## Disclosures

The authors report no conflict of interest concerning the materials or methods used in this study or the findings specified in this publication.

## Author Contributions

Primary surgeon: K Watanabe. Assistant surgeons: Yamashiro, R Ishikawa, Tomoto. Editing and drafting the video and abstract: Yamashiro, K Ishikawa. Critically revising the work: Yamashiro, N Watanabe, Matsuzawa. Reviewed submitted version of the work: K Watanabe, Yamashiro, Tomoto, K Ishikawa, Tanaka. Approved the final version of the work on behalf of all authors: K Watanabe. Supervision: K Watanabe, Murayama.

## Supplemental Information

### Patient Informed Consent

The necessary patient informed consent was obtained in this study.
